# Fitness to stand trial: 415 consecutive defendants assessed by a New Zealand forensic psychiatry service

**DOI:** 10.1177/10398562241290027

**Published:** 2024-10-07

**Authors:** Amber Wakefield, Susanna Every-Palmer, James A Foulds

**Affiliations:** Te Whatu Ora-Health New Zealand, 63588 Waitaha Canterbury, New Zealand; Department of Psychological Medicine, 8494 University of Otago Wellington, New Zealand; Department of Psychological Medicine, 2494 University of Otago Christchurch, New Zealand

**Keywords:** fitness to stand trial, competence to stand trial, forensic psychiatry

## Abstract

**Background:**

New Zealand defendants found unfit to stand trial following a Court-ordered forensic mental health assessment cannot be detained in prison and must either be released, or made subject to a mental health or intellectual disability order. There is increasing awareness of the need to identify these people and protect their rights.

**Methods:**

Retrospective audit of 8 years of Court-ordered health assessor reports addressing fitness to stand trial prepared by a New Zealand regional forensic mental health service with a catchment area of around 850,000.

**Results:**

Between 2014 and 2022, Courts referred 415 defendants for assessment of fitness to stand trial. The number of reports requested increased by 20% between 2014 and 2022. Report subjects were 81% male and had a median age of 31. Commonest primary diagnoses were psychotic disorders (37%), intellectual disability (13%) and acquired neurocognitive disorders (15%). Few people with foetal alcohol spectrum disorder were identified. Despite the increase in assessments, the number of defendants considered unfit by report writers remained stable over time.

**Conclusion:**

The increasing number of referrals for assessment of fitness to stand trial has resourcing implications for forensic mental health services.

Access to a fair trial is a fundamental human right.^1,2^ To exercise this right, defendants must have a basic understanding of the legal process and they must be able to help their legal counsel convey their decisions to the Court. Most international jurisdictions have mechanisms to assess whether a defendant is fit to stand trial. However, there remains concern about the extent to which mentally impaired people are treated fairly after they are charged with an offence.^3^ For example, 2007 UK data showed that while only 3% of people in prison had an intellectual disability, almost half had some impairment in communication.^4^ In New Zealand, a high profile 2015 appeal against a wrongful conviction for murder has drawn public awareness to protecting the rights of vulnerable defendants, particularly those with cognitive impairment.^5^

The statutory framework for fitness to stand trial in New Zealand (hereafter referred to as ‘fitness’) is set out in the Criminal Procedure (Mentally Impaired Persons) Act 2003 (‘CPMIP’). Paraphrasing Section 4 of this Act, a defendant is unfit to stand trial if they are unable, due to mental impairment, to: conduct a defence, or to instruct counsel; to plead; to adequately understand the nature, purpose, or possible consequences of the proceedings; or to communicate adequately with counsel to conduct a defence.^6^ Fitness issues can be raised by any of the parties to a Court proceeding, and they can be dealt with by either lower or higher Courts. Once found unfit, a defendant must be dealt with outside the criminal justice system. They can either be released outright or placed under an Order requiring treatment by mental health or intellectual disability services.

The 1836 English case *R v Pritchard*^7^ remains a landmark in British commonwealth jurisdictions like New Zealand. Concepts derived from *Pritchard* remain useful, but recent New Zealand case law such as *P* vs *Police [2006]*^8^ cites principles derived from the Australian case *R v Presser*.^9^ The Presser criteria are more expansive than *Pritchard*, with more focus on defendants’ understanding of their rights, and their ability to follow proceedings and make decisions in Court.^10^ The 2017 New Zealand Court of Appeal judgement in *Nonu v R*^11^ further extends these concepts derived from *Presser,* emphasising defendants’ capacity for *effective participation* in legal processes.

These recent case law developments in New Zealand seem to raise the bar for fitness. Indeed, a sharp increase in the number of unfit defendants was noted in the decade to 2018 ^12^ though recent New Zealand Ministry of Justice data show this spike may now have levelled off.^13^ Alongside this increased awareness of fitness issues, forensic mental health services are increasingly struggling to provide Court-ordered assessments^14^ and to provide care for defendants who have been found unfit to stand trial.^15^

There have been similar concerns about mentally impaired defendants in the United States. There, increased detection of vulnerable defendants has led to more referrals for competence assessment and restoration, leaving forensic mental health services struggling to keep up with demand.^16^ By contrast, fitness to stand trial continues to be raised infrequently in Australian jurisdictions,^17^ which has been criticised on human rights grounds.^18^

We hypothesised that increasing awareness of defendants with suspected mental impairment in New Zealand may have led to changes in the profile of people referred by courts for assessment of fitness. Quantifying this workload is vital considering the need for secure forensic hospital beds and the legal and human rights implications of failing to identify or provide care for unfit defendants.^14,15^ We aimed to examine 8-years trends in Court-ordered fitness assessments from a New Zealand regional forensic mental health service representing about one sixth of the New Zealand population.

## Methods

### Design

We did a retrospective audit of all Court-ordered health assessor reports addressing fitness to stand trial prepared by the Canterbury (New Zealand) regional forensic mental health service between 1 July 2014 and 30 June 2022. Measures were aggregated by year to investigate time trends. Ethical approval was received from the national health and disability ethics committee (reference 12,684) and the University of Otago Health Ethics Committee (reference HD22/105).

### Setting and participants

The Canterbury forensic mental health service is responsible for over 90% of the Court-ordered mental health reports for a catchment area of about 850,000 people (Canterbury, Nelson-Marlborough and West Coast). Reports were written by a psychiatrist or forensic-trained clinical psychologist (including two of the authors, AW and JF). When the question of fitness is raised, the CPMIP specifies that two assessors must prepare reports, but in practice, this sometimes does not occur. Where two reports are completed, assessors typically work independently but New Zealand High Court rules for expert witnesses require that, at the direction of the Court, experts must confer and try to reach agreement.

### Variables and data sources

One author searched the electronic database comprising all health assessor reports completed over the study period. All reports in which the Court had requested assessment of fitness to stand trial were included. Reports which solely addressed eligibility for an insanity defence or sentencing decisions were excluded. Some defendants had more than one report addressing fitness by each health assessor within the context of a single set of charges. This could arise when a defendant was initially judged unfit and later became fit with treatment. In these cases, only the findings from the last report of each health assessor were used.

Data extracted were: (a) age; (b) gender; (c) primary ethnicity; (d) the primary DSM-5 mental health diagnoses recorded in the report; (e) profession of the report writer (psychiatrist or clinical psychologist); and (f) the assessor’s opinion regarding fitness. In cases where two assessors’ primary diagnosis differed, an author reviewed the reports and relevant clinical records to assign one diagnosis. Data obtained from Court Orders were (a) the date the Order was received; (b) the offence category of the most serious of the current charges before the Courts, categorised according to the Australian and New Zealand Standard Offence Classification (ANSOC) system (2011). We also searched the electronic health records of unfit defendants to find their disposition by the Court.

### Statistical methods

Analyses were conducted in SPSS Version 28. Time trends in the number of defendants from 2014 to 2022 for whom reports were ordered and the number of defendants assessed as unfit to stand trial were analysed using linear regression.

## Results

During the study period, Courts requested fitness reports for 415 defendants, for whom 655 reports were completed. Psychiatrists completed 59% of reports and clinical psychologists wrote the remainder. In 105 cases (25%), at least one assessor judged the defendant to be unfit. Assessors disagreed about the defendant’s fitness to stand trial in eight cases. Of these, the Court found three defendants fit to stand trial, three were found unfit, and the outcome was unknown or not yet resolved in the two remaining cases. For a further two defendants, no clear opinion on fitness was expressed.

**Figure 1.**
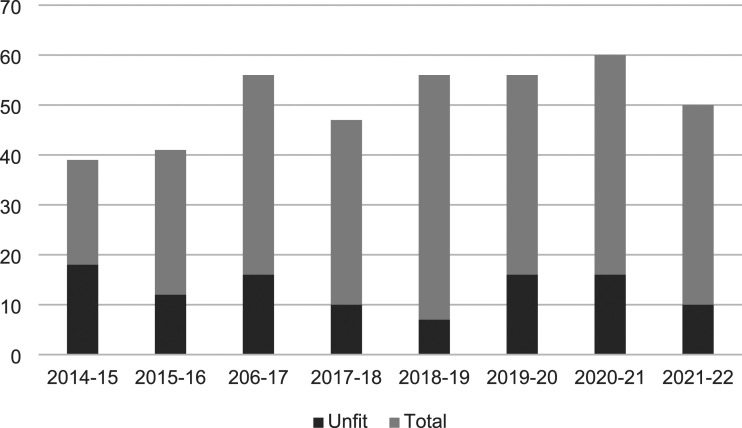
Number of people referred for assessment of fitness to stand trial and assessment outcomes 2014 to 2022. Legend: annual number of defendants referred for assessment of fitness to stand trial, and the number of those for whom at least one assessor judged the defendant unfit.

Figure 1 shows the annual number of reports and assessors’ opinions on fitness from 2014 to 2022. The number of defendants per year for whom assessments were requested ranged from 39 to 63 (median 49.5) The number of requests per year increased significantly (b = 2.54, *p* = 0.046) over the study period against a background of a 13% increase in the South Island population (see stats. govt.nz) and a 20% reduction in the number of people charged with offences in that time.^19^

Table 1 shows the demographic and clinical characteristics of the sample. Supplemental Tables S1 and S2 show these figures broken down by year. Psychotic disorders were the commonest primary diagnosis (*n* = 152; 37%), with over half of people in this group (*n* = 83; 20%) having a diagnosis of schizophrenia. Acquired neurocognitive disorders including those with traumatic brain injury or dementia (*n* = 61; 15%,) and congenital intellectual disabilities (*n* = 56; 13%) were the next commonest diagnostic groupings. A diagnosis of suspected or confirmed foetal alcohol spectrum disorder was recorded in six defendants. For 15% of defendants there was no primary mental disorder recorded in assessor report(s).

**Table 1.** Demographic and clinical characteristics of sample (*n* = 415)

	Number (%)
Gender (male)	336 (81%)
Ethnicity	
NZ European	225 (54%)
Māori	153 (37%)
Pasifika	11 (3%)
Asian	6 (1%)
Other	17 (4%)
Unknown or not recorded	3 (<1%)
Age (median, IQR)	31 (24-46)
Primary diagnosis	
Psychotic disorders^a^	152 (37%)
Intellectual disability	56 (13%)
Acquired neurocognitive disorders	61 (15%)
Mood disorders	27 (7%)
No diagnosis recorded	61 (15%)
Other^b^	58 (14%)

^a^Includes schizophrenia, schizoaffective disorder, delusional disorder, and psychotic disorder not otherwise specified.

^b^Includes posttraumatic stress disorder, attention deficit / hyperactivity disorder, autistic spectrum disorders, obsessive-compulsive disorder, substance use disorder and personality disorder.

Table 2 shows the classification of the most serious offence in the legal matter prompting fitness assessment. Violent offences including homicide, acts intended to cause injury, sexual offences, acts endangering persons and robbery made up 74% of cases.

**Table 2.** Offence Classification^a^ of most serious charge for people referred for assessment of fitness to stand trial

	Fit	Unfit	Total^b^
Homicide and related offences	15	2	17
Acts intended to cause injury	143	63	206
Sexual assault and related offences	36	15	51
Dangerous or negligent acts endangering persons	13	2	15
Abduction, harassment and other offences against the person	3	3	6
Robbery, extortion and related offences	10	2	12
Unlawful entry with intent/burglary, break and enter	25	6	25
Theft and related offences	11	2	11
Fraud, deception and related offences	2	3	5
Illicit drug offences	4	0	4
Prohibited and regulated weapons and explosives offences	8	1	9
Property damage and environmental pollution	11	3	14
Public order offences	1	0	1
Traffic and vehicle regulatory offences	7	1	8
Offences against justice procedures, government security and government operations	11	2	13
Totals	300	105	405

^a^Australian and New Zealand Standard Offence Classification.

^b^In 8 cases there was disagreement between assessors and in 2 cases no opinion on fitness was expressed. These cases have been excluded from the table above.

The time between a referral from the Court being received and the final assessment report being submitted to the Court ranged from 0 to 243 days (median 31 days). Some cases involving a long delay between the Court order and the final report were because the defendant initially unfit and became fit with treatment. In 46% of cases, the final report(s) requested by the Court (i.e. either one or both reports) had been submitted within 28 days of the Court Order.

Among the defendants who were considered unfit by a health assessor (*n* = 105), almost half had a diagnosis of intellectual disability (*n* = 43; 41%), followed by psychotic disorders (*n* = 32; 31%) and acquired neurocognitive disorders (*n* = 20; 19%).

The legal disposition was known for 76/105 defendants who were assessed as unfit. In the remaining 29 defendants the outcome was either not clearly recorded in health records or the matter was still before the Court at the time the study was completed. Thirty defendants were made subject to a Compulsory Care Order pursuant to intellectual disability legislation; 26 were ordered to be released without any treatment order; 15 were placed under a Compulsory Treatment Order pursuant to the Mental Health (Compulsory Assessment and Treatment) Act 1992; and five were placed under a forensic order as a special patient (*n* = 3) or intellectual disability special care recipient (*n* = 2).

## Discussion

There was a steady increase in Court referrals for assessment of fitness to stand trial between 2014 and 2022, despite a 20% reduction in the number of people charged with offences in New Zealand over that period.^19^ People referred for fitness assessment had a similar demographic profile to the population of people charged with offences.^19^ Most had an intellectual disability, an acquired neurocognitive disorder or a psychotic disorder, and about one quarter was assessed as unfit to stand trial. There was high concordance between assessors about a defendant’s fitness, possibly because rules for expert witnesses in New Zealand state experts must confer with each other at the direction of the Court, to try to reach agreement.

The increase in referrals for fitness assessment likely reflects increasing awareness of the needs of mentally impaired defendants following high-profile cases such as the case of Teina Pora.^5^ It may also reflect more mentally unwell people coming before the Courts. Ministry of Justice data for the whole of New Zealand showed the number of people found unfit to stand trial increased steeply between 2014 and 2017 before levelling off.^13^ In contrast to the national data, the number of people assessed as unfit in our sample remained relatively stable from 2014 to 2022, albeit with high variability from year to year. There is no clear reason why the Canterbury figures did not parallel what was happening in the rest of the country. However, the existence of a specialist Court in Auckland (but not other regions) to deal with suspect cases of unfitness to stand trial and defendants eligible for a defence of insanity^20^ could have contributed to regional differences. There is a need for further studies to examine these regional differences, and to show whether specialised courts need to be set up across the country.

Few cases of suspected foetal alcohol spectrum disorder (FASD) were identified in our sample. This is relevant as research suggests FASD is very common among people entering the justice system^21^ and disability advocacy groups identify people with FASD as needing special support during the court process.^22^ Our findings suggest FASD may be overlooked by Court-ordered fitness assessors, particularly when early medical records are unavailable. However, a focus on FASD may distract from the needs of a larger group of people whose cognitive impairment stems from other causes including severe childhood adversity, traumatic brain injury or substance use.

Court-ordered fitness assessments are an important mechanism to detect vulnerable defendants and ensure they get appropriate support, for example, a communication assistant. It is also vital to identify those who will regain fitness with treatment, as this group should have prioritised access to forensic inpatient care.^16^ One fifth of defendants who were assessed as unfit had an acquired neurocognitive disorder as their primary diagnosis. People in this group often present challenges for mental health services. They are less likely to respond to treatment than those with a psychotic disorder, therefore a treatment order pursuant to mental health legislation is often unhelpful. Intellectual disability legislation in New Zealand excludes people with an acquired neurocognitive disorder that began after the development period. There is a need for new legal pathways and better services for this group.

Forensic mental health services should identify potentially unfit defendants early in their legal proceedings regardless of their treatability. Early detection allows counsel to use their time more productively, and it helps avoid delays in legal proceedings and miscarriages of justice.^20^ When a defendant is suspected of being unfit, the forensic mental health service should follow them until the question of fitness is resolved. For those who are found unfit, follow up should continue at least until disposition by the Court has taken place.

Early detection of unfit defendants inevitably means having a low threshold for fitness assessment, though court liaison nurses can help with screening, which may avoid the need for some full assessments. Less than half of the fitness assessments in our sample were completed within 4 weeks, meaning that delays in legal proceedings may have occurred in many cases. There is a balance between ensuring unfit defendants are not missed, and subjecting defendants to assessments unnecessarily in a way that delays their time spent on remand. One solution is to ensure court liaison nurses are always available to the Courts, which has not always been the case in the outlying regions served by the Canterbury service. There also needs to be enough capacity among expert assessors who write reports. Recent media reporting has highlighted the problems the Courts face in obtaining these assessments.^14^ Forensic inpatient bed capacity is vital in cases where fitness is in doubt, or it can be restored with psychiatric treatment. This is difficult to achieve when specialist forensic services are under increasing pressure.^23^ The New Zealand Ministry of Justice has long recognised the importance of processes for assessing mentally impaired defendants. A dedicated Court for mentally impaired defendants has been operating in Auckland since 2000 ^20^, but this has not been expanded nationwide. In response to the well-publicised concerns about a lack of access to Court-ordered reports in some parts of New Zealand,^14^ the Ministry has recently set up a national network of expert assessors. Many of these reports are now being done privately rather than by forensic mental health services.

The limitations of the present study include incomplete data on disposition outcomes and relatively small numbers of unfit defendants per year, limiting the ability to detect shifts in the demographic and clinical profile of the sample over time. We also did not analyse the reasons assessors ascribed to their opinions on whether a defendant was fit or unfit, though this has previously been reported in a New Zealand sample.^24^ Most reports did not use a standardised fitness assessment instrument^17^ but this may not be necessary when assessors are applying well known criteria.

In summary, there was an increase between 2014 and 2022 in the number of defendants for whom Courts requested an assessment of fitness to stand trial, despite declining numbers of people charged with offences over that period. Detecting people who may be unfit to stand trial, and providing treatment to optimise their capacity to take part in Court proceedings, are core functions of forensic mental health services. It is vital that forensic mental health services continue to be resourced to do this work.

## Supplemental Material

Supplemental Material - Fitness to stand trial: 415 consecutive defendants assessed by a New Zealand forensic psychiatry service Supplemental Material for Fitness to stand trial: 415 consecutive defendants assessed by a New Zealand forensic psychiatry service by Amber Wakefield, Susanna Every-Palmer, James A Foulds in Australasian Psychiatry
