# Supplementary material for: Fitness to stand trial: 415 consecutive defendants assessed by a New Zealand forensic psychiatry service
Source: Australas Psychiatry. 2024 Oct 7;33(1):51–6. doi: 10.1177/10398562241290027 (PMC11804146; doi:10.1177/10398562241290027)
Supplement: Supplemental Material - Fitness to stand trial: 415 consecutive defendants assessed by a New Zealand forensic psychiatry service [file sj-pdf-1-apy-10.1177_10398562241290027.pdf]

Supplementary material

Table S1. Demographic data for Health Assessor reports addressing the issue of Fitness to Stand Trial from July 2014 to July 2023.

Demographic Factors	**2014-2015**	**2015-2016**	**2016-2017**	**2017-2018**	**2018-2019**	**2019-2020**	**2020-2021**	**2021-2022**	**Total**
Gender	*N*	*N*	*N*	*N*	*N*	*N*	*N*	*N*	*N (%)*
Male	31	30	48	32	47	53	50	45	336 (81%)
Female	8	11	10	15	9	6	13	7	79 (19%)
Ethnicity									
NZE	23	26	36	27	26	25	36	26	225 (54%)
Māori	12	14	15	17	22	30	21	22	153 (37%)
Pacifica	1	-	2	-	2	3	2	1	11(3%)
Asian	-	-	1	1	2	-	1	1	6 (1%)
Other	3	1	4	2	2	1	2	2	17 (4%)
Unknown	-	-	-	-	2	-	1	-	9(2%)
Age									
<19	4	7	1	5	2	4	3	2	28 (7%)
20-24	11	12	15	12	11	8	12	5	86 (21%)
25-29	7	8	7	6	15	8	10	10	71 (17%)
30-34	6	6	6	5	2	12	12	13	62 (15%)
35-39	1	-	4	2	5	10	5	6	33 (8%)
40-44	2	2	5	7	10	7	3	4	40 (10%)
45-49	1	2	8	4	2	3	3	4	27 (7%)
50-54	3	-	5	3	6	3	3	2	25 (6%)
55-59	1	2	3	-	2	4	5	2	19 (5%)
60-64	1	1	2	1	1	-	4	2	12 (2%)
>65	2	1	2	2	-	-	3	2	12 (2%)
Total	**39**	**41**	**58**	**47**	**56**	**59**	**63**	**52**	**415**

Table S2. Diagnosis of those undergoing court ordered reports addressing the issue of fitness July 2014 to July 2022 [a]

Diagnosis	**2014-2015**		**2015-2016**		**2016-2017**		**2017-2018**		**2018-2019**		**2019-2020**		**2020-2021**		**2021-2022**		**Totals**
	Unfit	*Fit*	*Unfit*	*Fit*	*Unfit*	*Fit*	*Unfit*	*Fit*	*Unfit*	*Fit*	*Unfit*	*Fit*	*Unfit*	*Fit*	*Unfit*	*Fit*	*N (%)*
No mental impairment	-	1	-	6	-	14	-	9	-	1	-	13	-	11	-	4	61 (15)
Intellectual Disability	5	-	3	3	6	2	5	1	3	1	9	-	8	1	4	2	56 (13)
Cognitive Impairment	3	3	1	2	3	5	4	5	2	5	1	4	4	7	2	8	61 (15)
Asperger's	-	1	2	-	1	-	-	-	-	1	-	2	-	2	1	-	10 (3)
ADHD	-	-	-	1	-	1	-	1	-	2	-	-	-	2	-	3	10 (3)
Delusional disorder	1	-	1	-	1	-	-	-	-	-	-	1	-	1	-	-	5 (1)
Schizoaffective	1	-	2	5	2	2	-	-	-	2	-	2	-	2	-	6	25 (6)
Schizophrenia	3	5	1	5	2	6	1	8	2	11	5	7	3	9	2	11	83 (20)
Psychosis NOS	3	4	1	1	1	4	-	4	-	7	-	5	-	6	-	3	39 (9)
Mood Disorder	2	1	-	2	-	2	-	4	-	6	1	5	1	1	1	1	27 (7)
OCD	-	-	-	1	-	-	-	-	-	-	-	-	-	-	-	-	1 (<1)
PTSD	-	-	-	-	-	2	-	-	-	1	-	1	-	1	-	-	5 (1)
Somatic Disorder	-	1	-	-	-	-	-	-	-	-	-	-	-	-	-	-	1 (<1)
Substance Use Disorder	-	4	-	-	-	1	-	2	-	5	-	-	-	-	-	2	14 (3)
Personality disorder	-	1	1	3	-	1	-	3	-	7	-	-	-	1	-	-	17 (4)

Total	**18**	**21**	**12**	**29**	**16**	**40**	**10**	**37**	**7**	**49**	**16**	**40**	**16**	**44**	**10**	**40**	**405**

a. 8 cases in which there was disagreement between assessors about fitness and 2 cases in which no opinion was expressed on fitness have been excluded from the above table.
